# Insulin signaling pathways in a patient with insulin resistance of difficult management - a case report

**DOI:** 10.1186/1758-5996-1-23

**Published:** 2009-11-26

**Authors:** Giselle F Taboada, Marta S de Freitas, Fernanda H da S Corrêa, Carlos RMA Junior, Marília de B Gomes

**Affiliations:** 1 Diabetes & Metabology Unit, Internal Medicine Department, State University of Rio de Janeiro, Rio de Janeiro, Brazil; 2 Pharmacology Department, Biology Institute, State University of Rio de Janeiro, Rio de Janeiro, Brazil

## Abstract

Insulin signalling pathways were investigated in a 33 year-old woman with immunologic insulin resistance. Her past medical history was remarkable for intermittent use of insulin and allergic reactions to several drugs, and measure of plasma anti-insulin antibodies level corroborated the clinical suspicion of immune mediated insulin resistance (8074 nU/ml - RIA - Ref value: <60). Treatment with several immunosuppressive regimens was tried, however the results were disappointing. Possible subcellular mechanisms of insulin resistance were investigated by performing analysis of insulin receptor and post receptor signaling in skeletal muscle biopsy. The expression of insulin receptor (IR), insulin receptor substrate 1 (IRS-1) and glucose transporter 4 (GLUT-4) was evaluated in total extract from muscle tissue by Western blotting. Basal IR, IRS-1 and GLUT-4 expression was detected, however receptor autophosphorylation was not observed. A study of translocation of GLUT-4 to plasma membrane showed that tissue presented low levels of membrane-associated GLUT-4. When *in vitro *stimulation was undertaken, tissue was capable to be responsive to insulin. Our results suggest that even though IR expression was normally occurring, IR β-subunit tyrosine kinase activity in muscle was down-regulated leading to alterations in insulin post receptor signaling. Consistent with normal insulin receptor and post receptor signaling, our results were compatible with decreased insulin binding to IR probably due to neutralization by anti-insulin antibodies. In conclusion, this patient has immunologic insulin resistance and treatment should be based on immunosuppressive drugs as tolerated.

## Introduction

Immunologic insulin resistance is a disorder recognized in the clinical practice for many years [1,2]. Its pathogenesis is related to the presence of plasma anti-insulin antibodies, and patients at greatest risk are those who ever used non-human insulin and those who had a history of intermitent use of this drug [3]. Treatment interruption and re-introduction of insulin works as a booster, a very well-known effect in the immunization practice that induces the rising of antibodies against the presented antigen. Other patients at risk for the development of anti-insulin antibodies are those with an allergic history or with auto-immune diseases [4].

After excluding the possibility of resistance to the subcutaneous route of administration by using the parenteral route [5] and making a trial of a synthetic insulin analogue [6], treatment is based upon immunosuppression. Many regimens have already been described as resulting in good clinical and laboratorial results but there are no consensus in the literature as to which is the best one [3-5,7].

We hereby present a case of immunologic insulin resistance that has not responded to many therapeutic immunosuppressive interventions resulting in great anxiety for assistant physicians and leading us to investigate the possibility of a subcellular mechanism of disease.

## Case Report

A 33 year-old woman with diabetes for 25 years was treated with NPH insulin (0.77 U/kg/day) when she first came to our attention. She had a positive history of allergy to penicillin and no other morbidities. During her adolescence, she had been switched to oral hypoglycemiants for approximately 4 years, which made her diabetes difficult to classify.

During follow-up we were able to gradually decrease her insulin dose until she was successfully switched back to oral agents. For 11 months she was on a regimen of glyburide (10 mg bid), metformin (850 mg tid) and acarbose (50 mg tid), before being admitted with hyperosmolar non-ketotic coma.

After a prolonged hospital stay she was discharged home using NPH insulin. Over the following months, worsening glycemic control indicated progressive insulin dose increments (from 0.6 to 5.7 U/Kg/day). When 2.7 U/Kg/day was reached, we discarded all causes of secondary diabetes and plasma anti-insulin antibodies were determined (8074 nU/ml - RIA - Ref value: <60). We concluded she developed immunologic insulin resistance.

Several immunosuppressive regimens were tried such as glucocorticoids [7], cyclophosphamide [5], immunoglobulin [8] and plasmapheresis [5,7] but the results were disappointing (average glycemia before: 468 mg/dl, insulin dosage: 490 U/d; average glycemia after: 450 mg/dl, insulin dosage: 450 U/d) and life-threatening infectious complications indicated immunosuppressant interruption every time.

Subcutaneous resistance to insulin was ruled out by intravenous administration during the inpatient period [5]. Thereafter the patient was switched to Lispro [6] administered by continuous subcutaneous infusion.

**Figure 1:**
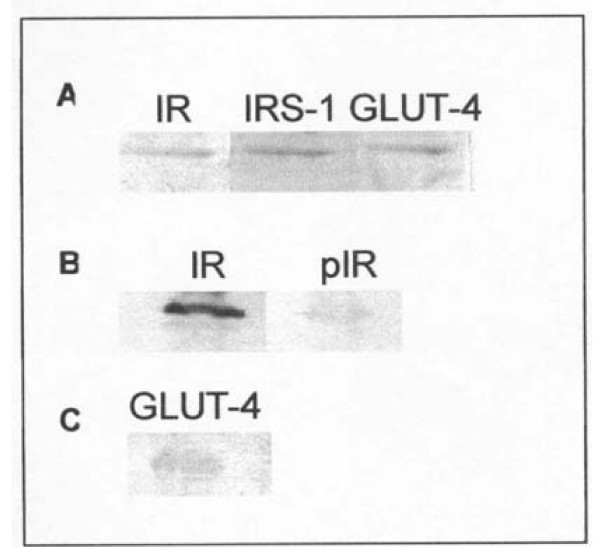
**A- IR, IRS-1 and GLUT-4 expression in total extract from skeletal muscle; B- IR protein content and IR tyrosine phosphorylation (pIR); C- Membrane-associated GLUT-4 content**. Cellular proteins (30 μg total) were subjected to 10% SDS-PAGE, transferred to PVDF filters and blocked with Tween-TBS containing 1% BSA. Primary antibodies used in Western analysis were anti-insulin receptor β-subunit (IR); anti-IRS-1; anti-GLUT-4 and anti-phosphotyrosine. PVDF filters were next incubated with appropriate secondary antibody conjugated to biotin followed by 1-h incubation with horseradish peroxidase-conjugated streptavidin. Immunoreactive proteins were visualized by DAB staining.

**Figure 2:**
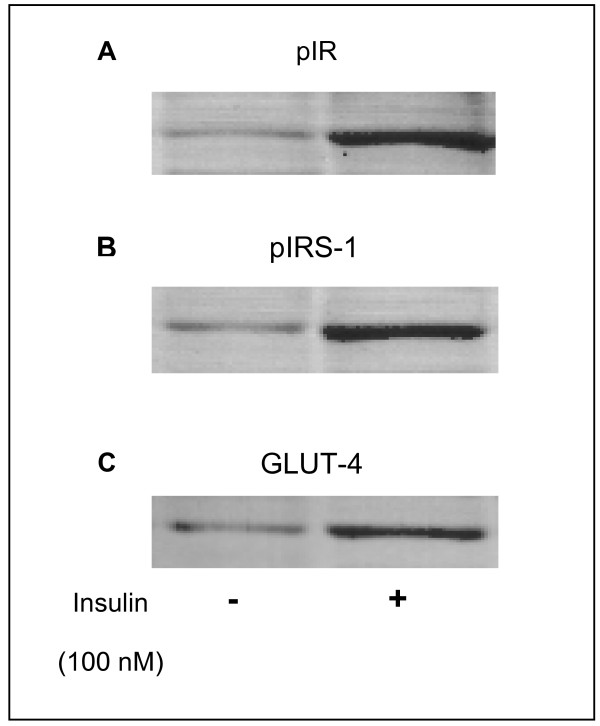
**A- IR phosphotyrosine content (pIR); B- IRS-1 phosphotyrosine content (pIRS-1) and C- Membrane-associated GLUT-4 content before (-) and after (+) *in vitro *insulin stimulation**. Skeletal muscle strips were stimulated with 100 nM insulin for 30 minutes and Western blottings were next probed with anti-phosphotyrosine antibody.

Since we had no success with the various immunosuppressive regimens we decided to perform a skeletal muscle biopsy to investigate possible subcellular mechanisms of insulin resistance. The patient gave informed consent for the procedure and it was accepted by the local ethics committee. Solear muscle was biopsied under topical anesthesia one hour after the patient administered a subcutaneous insulin bolus followed by a meal. Skeletal muscle membranes were isolated as described by Ozanne et al [9]. Expression of insulin receptor (IR), insulin receptor substrate 1 (IRS-1) and glucose transporter 4 (GLUT-4) was evaluated in total extract from muscle tissue by Western blotting as previously described by De Freitas et al [10]. Figure 1A shows basal IR, IRS-1 and GLUT-4 expression. However, when IR tyrosine phosphorylation (pIR) was analyzed, receptor autophosphorylation was not observed (Figure 1B) despite the expressive IR expression. Translocation of GLUT-4 to plasma membrane was also investigated in skeletal muscle. Western blotting protein analysis with anti-GLUT-4 antibody indicated that tissue presented low levels of membrane-associated GLUT-4 (Figure 1C). These findings suggest that even though IR expression was normally occuring, IR β-subunit tyrosine kinase activity in muscle was down-regulated leading to alterations in insulin post receptor signaling. According to this, IR (pIR) and IRS-1 (pIRS-1) phosphotyrosine contents and GLUT-4 translocation were analyzed by immunoblotting before and after *in vitro *stimulation with insulin. We found that IR and IRS-1 tyrosine phosphorylation was highly stimulated by insulin (Figures 2A and 2B) and GLUT-4 translocation was significantly increased (Figure 2C). Consistent with normal insulin receptor and post receptor signaling, our results were compatible with decreased insulin binding to IR probably due to neutralization by anti-insulin antibodies. Tissue was showed to be normally responsive to insulin and the failure in its action was probably due to the presence of plasma anti-insulin antibodies.

Once insulin dose still needed to be increased, patient was started on a combination of micophenolate mophetil (1 g bid) and prednisone (40 mg/d) in an outpatient basis. After one year, insulin dose was reduced from 5.8 to 3.48 U/Kg/d and glycemic control, even if not optimum, has shown some improvement (average glycemia: 390 to < 200 md/dL). It is important to note that patient did not experience fasting hypoglycemia since severe insulin resistance developed.

We conclude that this patient has immunologic insulin resistance of difficult management. The best immunosuppressive regimen for her, in terms of improvement in glycemic control, was the combination of prednisone and micophenolate mophetil, however the results are slow as a result of the type of response to these drugs. The muscular biopsy helped to rule out a subcellular mechanism of insulin resistance that would be more difficult to treat.

## Competing interests

The authors declare that they have no competing interests.

## Authors' contributions

GFT, FHSC and CRMAJ drafted the manuscript and were responsible for patient's medical assistance, MSF carried out the molecular genetic studies, GFT, MSF and MBG participated in the design of the study. MBG coordinated the study. All authors read and approved the final manuscript.
